# Supplementary material for: PAK1 as a Potential Therapeutic Target in Male Smokers with EGFR-Mutant Non-Small Cell Lung Cancer
Source: Molecules. 2020 Nov 27;25(23):5588. doi: 10.3390/molecules25235588 (PMC7729550; doi:10.3390/molecules25235588)
Supplement: Supplementary file 1 [file molecules-25-05588-s001.pdf]

Appendix A

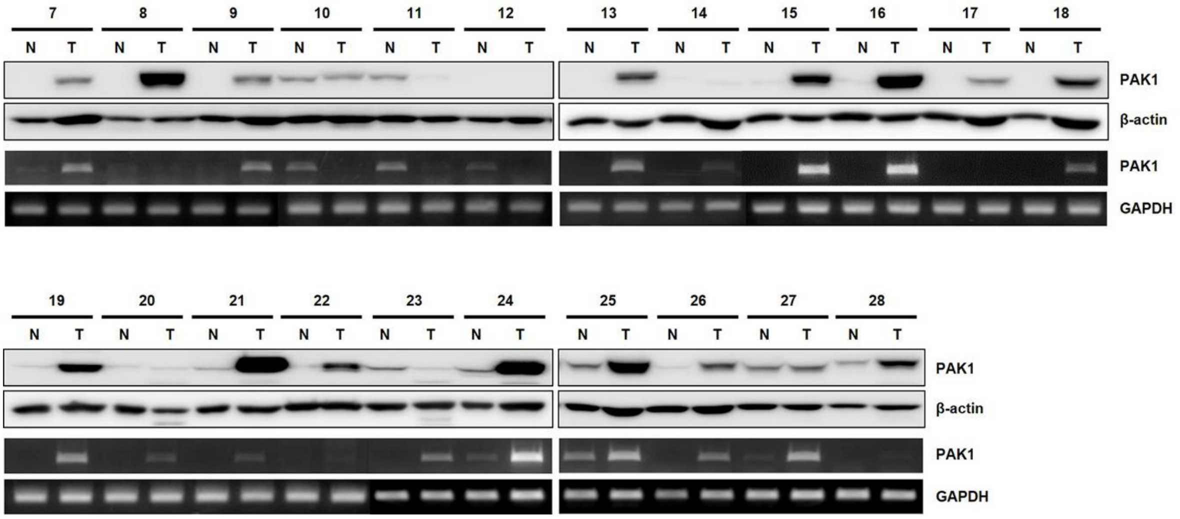

Supplementary Figure 1. PAK1 mRNA and protein level of PAK1 in human NSCLC and adjacent healthy tissues. Expression levels of both PAK1 mRNA and protein were higher in frozen NSCLC tissue specimens than that in the adjacent healthy tissue specimens from 28 NSCLC patients.

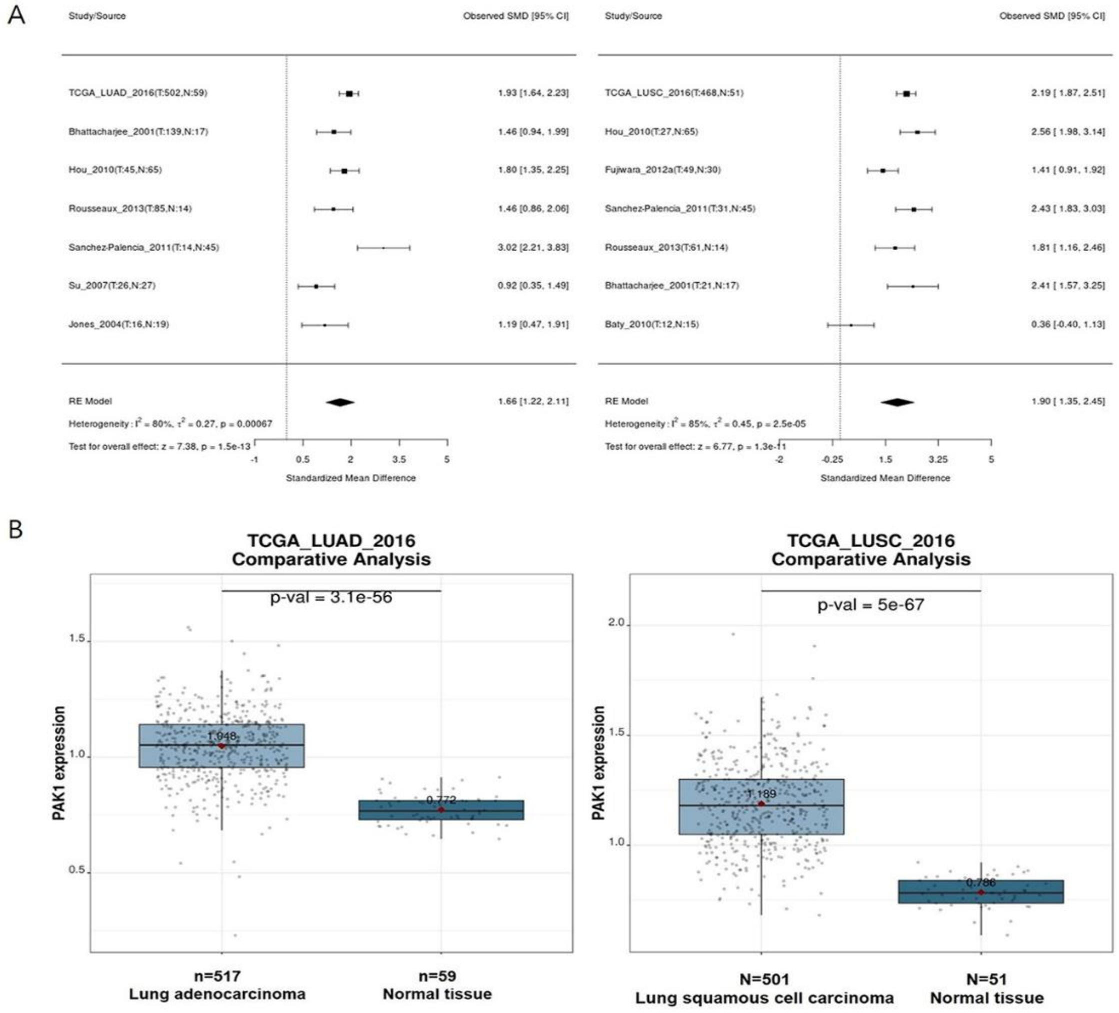

Supplementary Figure 2. Meta-analysis and comparative analysis of PAK1 expression between NSCLC and healthy tissues. (A) Meta-analysis and (B) comparative analysis of PAK1 expression revealed that PAK1 expression was higher compared with samples from healthy tissues.

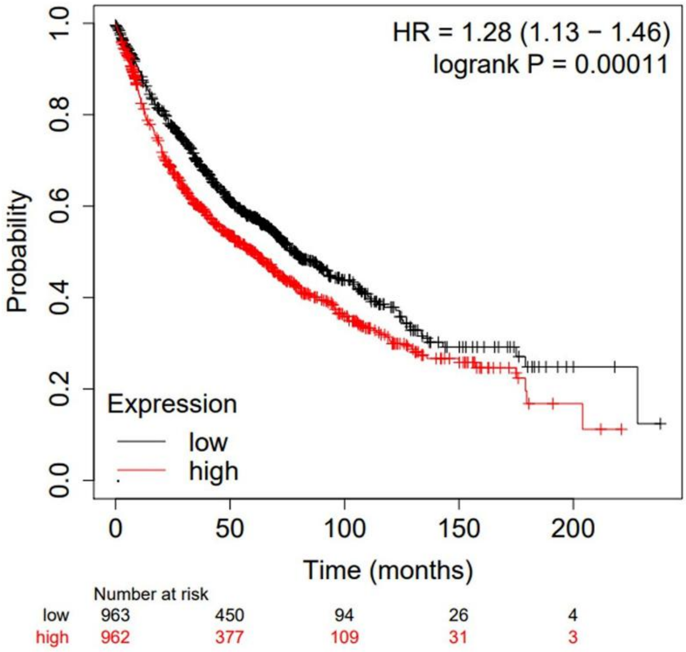

Supplementary Figure 3. Kaplan-Meier survival curves of PAK1 based on publicly available big data. Overexpressed PAK1 mRNA levels were associated with poor overall survival of lung cancer patients.

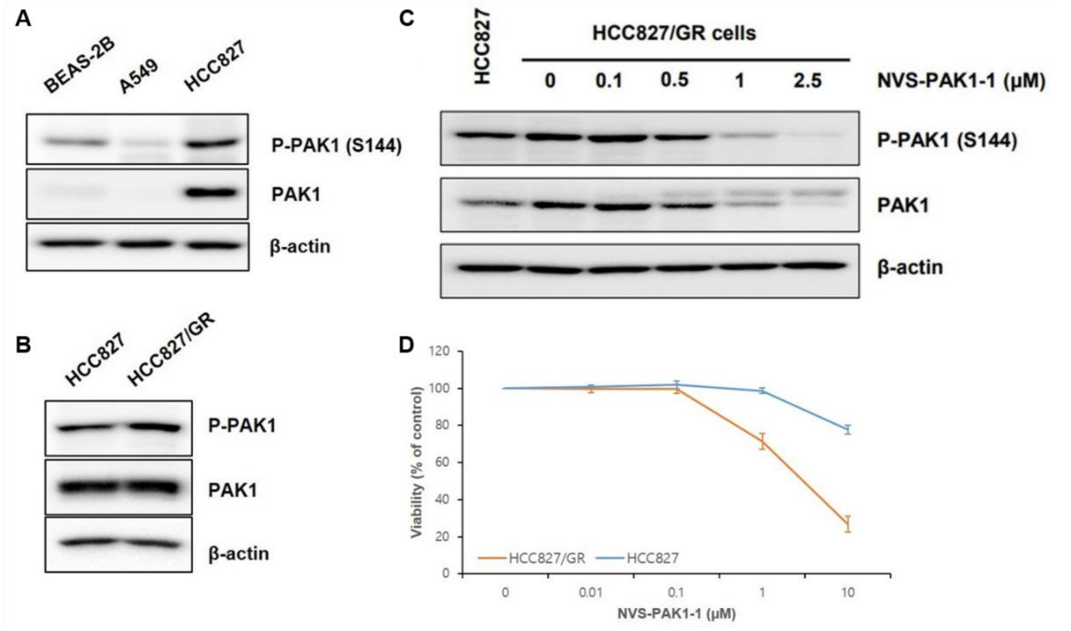

Supplementary Figure 4. Inhibitory effects of a PAK1 selective inhibitor, NVS-PAK1-1, on EGFR tyrosine kinase inhibitor-resistant lung cancer cell growth. (A) PAK1 and phospho-PAK1 levels were elevated in EGFR-mutant cell line, HCC827 in HCC827 (EGFR-mutated adenocarcinoma cell line) cells compared to those in A549 (EGFR wild-type cell line) or BEAS-2B (normal bronchial epithelial cell line) cells (B) geftinib-resistant HCC827 (HCC827/GR) cells had higher levels of phosphorylated PAK1 than wild-type HCC827 cells (C) Suppression of the PAK1 and phospho-PAK1 levels by NVS-PAK1-1 in HCC827/GR cells in a dose-dependent manner (D) Inhibitory effects of NVS-PAK1-1 on HCC827/GR growth
